# Circular RNA CircHIPK3 Elevates CCND2 Expression and Promotes Cell Proliferation and Invasion Through miR-124 in Glioma

**DOI:** 10.3389/fgene.2020.01013

**Published:** 2020-08-28

**Authors:** Zengjin Liu, Shewei Guo, Hongwei Sun, Yahui Bai, Zhenyu Song, Xianzhi Liu

**Affiliations:** Department of Neurosurgery, The First Affiliated Hospital of Zhengzhou University, Zhengzhou, China

**Keywords:** circular RNA, glioma, miR-124, circHIPK3, proliferation, invasion

## Abstract

As a malignant tumor of the central nervous system, glioma exhibits high incidence and poor prognosis. Circular RNA HIPK3 (circHIPK3) is a circular RNA (circRNA) related to cancer progression. However, the role of circHIPK3 in gliomas remains unclear. The purpose of this study was to investigate the role of circHIPK3 in gliomas and its mechanism. The qRT-PCR method was used to determine the expression pattern of circHIPK3 in glioma cell lines. CCK-8 assay was used to detect cell proliferation. Cell migration and invasion were evaluated using the Transwell assay. Our results showed that circHIPK3 expression was significantly up-regulated in glioma tissues and cell lines. *In vitro*, the down-regulation of circHIPK3 significantly inhibited the proliferation, migration and invasion of glioma cells. Besides, we demonstrated that circHIPK3 acted as a sponge to absorb miR-124 and promoted CCND2 expression. In summary, our results indicated that circHIPK3 had carcinogenic effects by regulating the expression of CCND2 in glioma by sponging miR-124. These findings provided favorable evidence to reveal the role of circHIPK3 in the development of gliomas.

## Introduction

Glioma is the most severe primary human central nervous tumor in adults worldwide, accounting for 40% of intracranial tumors, and has a poor prognosis (Geng et al., 2015). The growth and metastasis of glioma cells depend on angiogenesis, and a continuous increase in blood vessels has been considered a key feature of gliomas (Lund et al., 1998). This characteristic of rapid growth and high infiltration causes most patients already at stage IV when they are diagnosed with glioma (Shraddha et al., 2015). Pathological diagnosis is the basis for treatment. When considering the prognosis of osteosarcoma, a biopsy is performed to obtain confirmation by pathological examination as soon as possible, which is of great significance for the diagnosis and treatment. Despite the rapid development of science and technology in recent years, the comprehensive treatment of gliomas (including surgical resection, radiotherapy and chemotherapy) has also progressed, the treatment effect is still not ideal and the patient survival rate has not improved (Johnson and Sampson, 2010). Due to the special pathological and physiological characteristics of gliomas, there is currently no targeted treatment for them (Van Meir et al., 2010). In order to overcome the existing challenges, efforts need to be focused on developing new therapies for gliomas.

Circular RNA (circRNA) is a recently discovered class of endogenous non-coding RNA (ncRNA) that usually originates from exon regions, so it is also defined as exonic circRNA, but it may also arise from the intron and intergenic regions. It is proposed as an important regulator for understanding growth and development, tissue regeneration, potential pathological mechanisms and therapeutic targets for diseases, as it can transcriptionally or post-transcriptionally modulate gene expression by regulating microRNAs or other molecules. CircRNA sponges miRNAs to further regulate downstream gene expression (Ebbesen et al., 2016, 2017). For example, Yuan et al. (2019) showed that circRNA_005647 was upregulated in cardiac fibrosis and inhibited the expression of fibrosis-related genes through sponging miR-27b-3p in mouse CFs. Increasing evidence suggests that dysregulation of circRNAs plays a key role in the pathogenesis of many human diseases, such as malignancies.

CircHIPK3, produced by reverse splicing of the second exon of the HIPK3 gene, was reported to enhance cell growth and metastasis via binding and inhibiting many tumor-suppressive miRNAs (Zheng et al., 2016). For instance, there are reports indicating that circHIPK3 is overexpressed in prostate cancer tissues and is associated with tumor stage (Chen et al., 2019). Chen et al. (2018) found that circHIPK3 promoted cell proliferation and migration of liver cancer cells. Although circHIPK3 has been shown to increase expression in glioma tissues and can be used as a prognostic biomarker, the potential mechanism of circHIPK3 in gliomas remains unclear and requires further study (Jin et al., 2018).

MicroRNAs are endogenous short non-coding RNA molecules that negatively regulate gene expression at a post-transcriptional level. MiR-124 is one of the most abundantly expressed miRNAs in the brain that participates in the process of neurogenesis, synapse morphology, neurotransmission, inflammation and so on. Accumulating evidence shows that miR-124 plays an indispensable role in the progression of multiple diseases. For example, An et al. (2017) reported that miR-124 acted as a target for Alzheimer’s disease by regulating BACE1.

CCND2 is the abbreviation of cyclin D2. The protein encoded by this gene belongs to the highly conserved cyclin family, whose members are characterized by a dramatic periodicity in protein abundance through the cell cycle (Witt et al., 2013). Cyclin D has been shown in many cancer types to be misregulated. CCND2 has been the focus of major research and development efforts over the past decade. For example, Wang et al. found that lncRNA KCNQ1OT1 acting as a ceRNA for miR-4458 enhanced osteosarcoma progression by regulating CCND2 expression. All in all, the roles of miR-124 and CCND2 in glioma still keep unclear (Wang et al., 2019).

## Materials and Methods

### Clinical Samples and Cell Culture

This study was approved by the Medical Ethical Committee of the First Affiliated Hospital of Zhengzhou University. Glioma tissues and normal tissues were obtained from the First Affiliated Hospital of Zhengzhou University between October 2015 and September 2018. We obtained the informed consent from all patients. SW1783, and U373 were purchased from the TCCCAS (Shanghai, China). RPMI-1640 medium with 10% FBS, 1% penicillin/streptomycin was used to culture cells. All regents were purchased from Hyclone (Hyclone, Logan, UT).

### Cell Transfection

SW1783 and U373 cells in the logarithmic growth were transfected with 100nM si-circHIPK3/NC, the siRNA oligo was synthesized by Shanghai GenePharma Co., Ltd. The sequence of the siRNA for the circHIPK3 was 5′- CUACAGGUAUGGCCUCACA-3′ (si-circHIPK3). The sequence of negative control siRNA (si-NC) was 5′-UUCUCCGAACGUGUCACGUTT-3′. miR-124/NC inhibitor, miR-124/NC mimics, CCND2/empty vector plasmid (Genepharma, Shanghai, China) using Lipofectamine2000 (Invitrogen, CA) according to the manufacturer’s instructions.

### Cell Proliferation Assay

CCK-8 kit (Beyotime, Shanghai, China) was applied to detect cell viability at 0, 1, 2, 3, or 4 days after transfection. Briefly, 2000 cells/well glioma cells were seeded into 96-well plates. CCK-8 assay was detected using the microplate reader (Bio-Tek, Winooski, VT) according to the manufacturer’s instructions.

### Cell Migration and Invasion Assays

Cell metastasis was detected using Transwell chambers. For the invasion assay, chambers were coated with Matrigel. After transfection, the transfected cells were plated in the upper chamber with 1 × 10^5^ cells/well, while the lower chamber was filled with complete medium. One day later, the cells were fixed with methanol and stained with DAPI (Solarbio, Beijing, China) for 10 min.

### qRT-PCR

RNAs were extracted using TRIzol reagent. Reverse transcription of 2μg RNA was conducted using SuperScript RT kit. All reagents used in this section were purchased from Invitrogen (Invitrogen, Carlsbad, CA, United States). qRT-PCR was conducted using SYBR Premix ExTaq^TM^ with an Applied Biosystems 7300 system (Applied Biosystems, CA, United States). GAPDH or U6 was used as internal references to determine the relative expressions of targets by using the 2^–ΔΔ*Ct*^ method. The primers were purchased from Genepharm (Shanghai).

### Targets Prediction

We aimed to construct a glioma specific circHIPK3 regulating ceRNA network following several steps. First, we identified survival related genes in gliomas using GSE33331 database, which included 13 glioma samples with short overall survival time and 13 glioma samples with long overall survival time. Second, we identified circHIPK3 binding miRNAs using circBase database. Third, we predicted the potential targets of circHIPK3 binding miRNAs with miRTarbase and Targetscan. Finally, the ceRNA network was constructed using Cytoscape software.

### Gene Ontology and Pathway Enrichment Analyses

The Gene ontology and pathway enrichment analyses were conducted using DAVID system^1^.

### Luciferase Reporter Assay

The fragments of circHIPK3 and CCND2 containing the wildtype or mutant miR-124 binding sites were purchased from Sangon Biotech (Shanghai, China), and sub-cloned into psiCHECK-2 system (Promega, Madison, WI, United States). The luciferase activity was measured using the dual-luciferase reporter assay system (Promega) according to the manufacturer’s instructions.

### Statistical Analysis

The Student’s *t*-test was used for the statistical analysis. The SPSS software (version 20.0, SPSS, Inc., Chicago) was applied for the data analysis. *P* < 0.05 was considered statistically significant.

## Results

### Construction of CircHIPK3-RNA Binding Protein (RBP) Interaction Network

**FIGURE 1:**
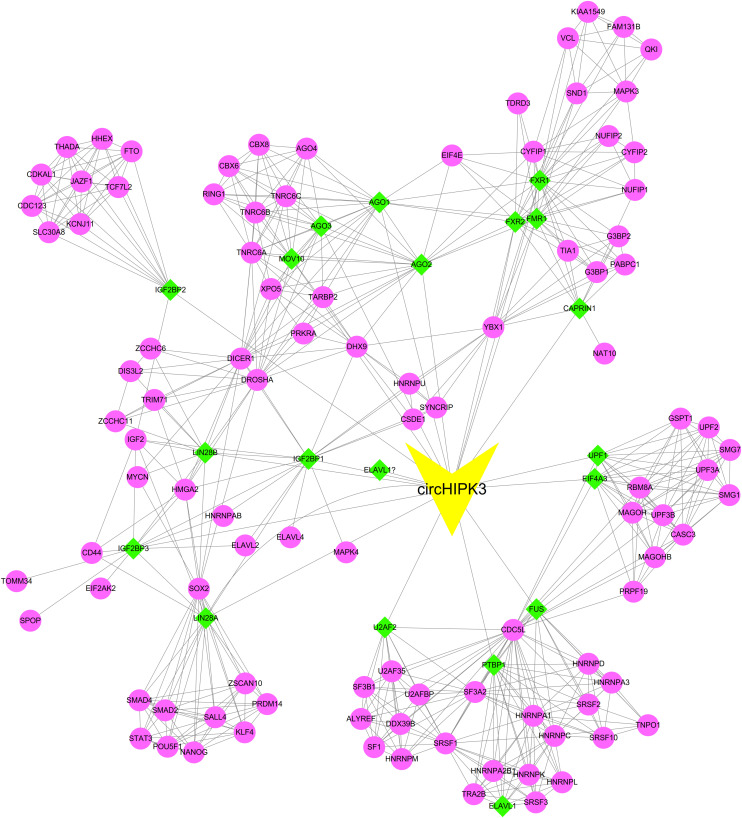
Construction of circHIPK3-RNA binding protein (RBP) interaction network circHIPK3 interacted with 20 RBPs.

In order to comprehensively predict the roles of circHIPK3 in human cancers, we first predicted the interacting RBPs of this circRNA using Circular RNA Interactome^2^. We found that circHIPK3 interacted with 20 RBPs, including AGO1, AGO2, AGO3, CAPRIN1, EIF4A3, ELAVL1, FMR1, FUS, FXR1, FXR2, IGF2BP1, IGF2BP2, IGF2BP3, LIN28A, LIN28A, LIN28B, MOV10, PTBP1, U2AF2, UPF1. Moreover, we constructed a complicated protein-protein interaction network to reveal the potential binding proteins of circHIPK3 interacting RBPs. As presented in Figure 1, the network included 1 circRNA, 121 proteins and 605 edges.

### Construction of CircHIPK3 Regulating ceRNA Network in Glioma

**FIGURE 2:**
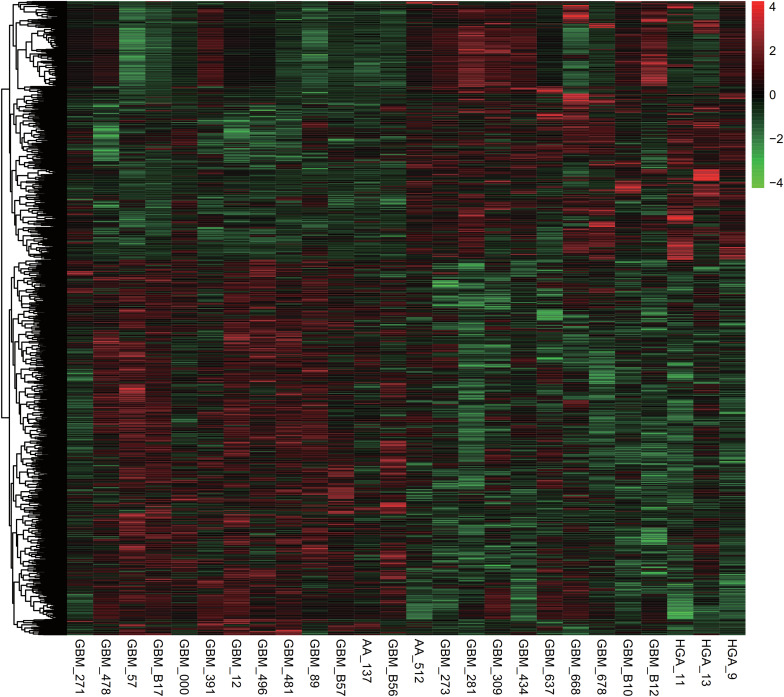
Survival related genes in gliomas were identified using the GSE33331 database of the 955 survival related genes identified in gliomas, 432 genes were related to long survival time, and 523 genes were related to short survival time.

Over the past decade, several previous studies showed circHIPK3 could acted as miRNA sponges to affect multiple genes’ expression. Very interestingly, we observed circHIPK3 could interacted with AGO1, AGO2, and AGO3, which were reported to be crucial regulators of miRNAs’ activity. Thus, we aimed to construct a glioma specific circHIPK3 regulating ceRNA network following several steps. First, we identified survival related genes in gliomas using GSE33331 database, which included 13 glioma samples with short overall survival time and 13 glioma samples with long overall survival time. As shown in Figure 2, a total of 955 survival related genes were identified in glioma. Among them, 432 genes were found to be related to long survival time and 523 genes were found to be related to short survival time. Second, we identified circHIPK3 binding miRNAs using circBase database. Third, we predicted the potential targets of circHIPK3 binding miRNAs. Finally, the ceRNA network was constructed using Cytoscape software.

**FIGURE 3:**
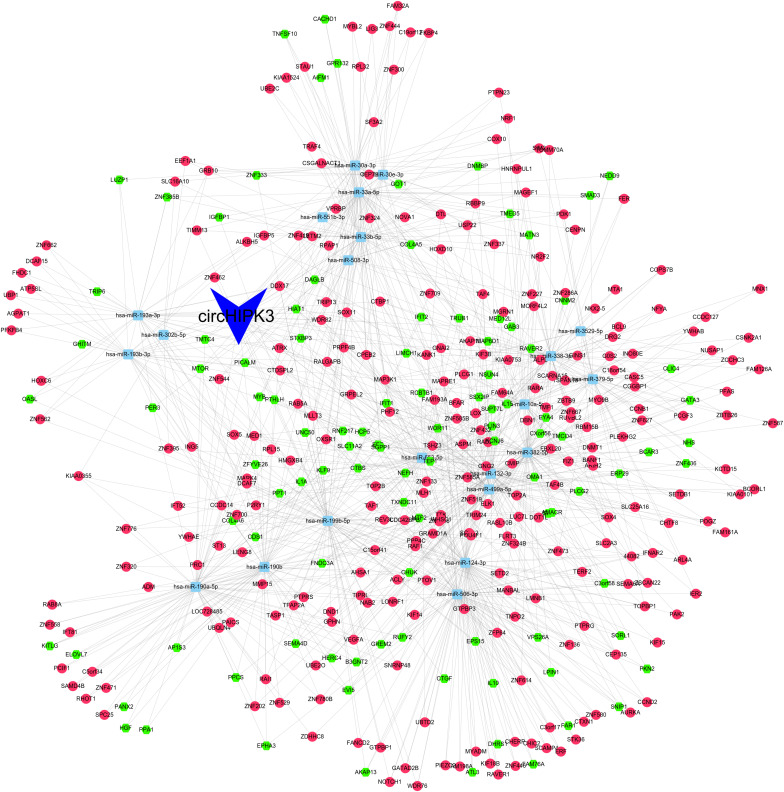
The ceRNA network was constructed using Cytoscape software the ceRNA network included 22 miRNAs and 372 mRNAs.

As presented in Figure 3, the ceRNA network included 22 miRNAs (hsa-miR-190b, hsa-miR-382-5p, hsa-miR-338-3p, hsa-miR-499a-5p, hsa-miR-124-3p, hsa-miR-33a-5p, hsa-miR-506-3p, hsa-miR-653-5p, hsa-miR-190a-5p, hsa-miR-379-5p, hsa-miR-199b-5p, hsa-miR-10a-5p, hsa-miR-193b-3p, hsa-miR-193a-3p, hsa-miR-508-3p, hsa-miR-33b-5p, hsa-miR-132-3p, hsa-miR-30a-3p, hsa-miR-30e-3p, hsa-miR-551b-3p, hsa-miR-3529-5p, hsa-miR-302b-5p) and 372 mRNAs.

### Bioinformatics Analysis of CircHIPK3 in Glioma

**FIGURE 4:**
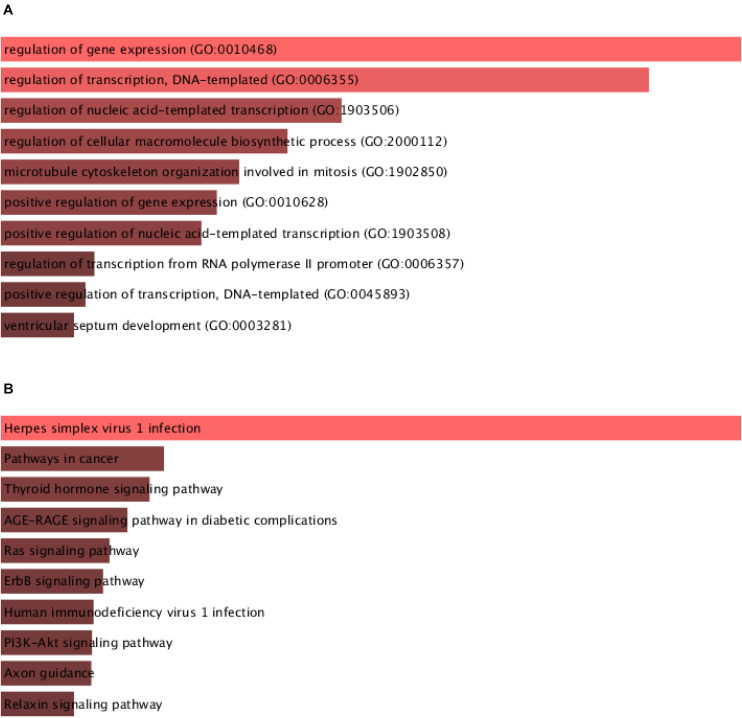
Bioinformatics analysis of circHIPK3 in glioma.**(A)** GO analysis of circHIPK3 in gliomas showed that it was involved in the regulation of gene expression and transcription. **(B)** Analysis of the KEGG pathway of circHIPK3 showed that it is involved in regulating Thyroid hormone signaling, Ras pathway, and ErbB signaling.

Then, we predicted the potential functions of circHIPK3 in glioma using ceRNA network. The GO analysis indicated that circHIPK3 was involved in regulating gene expression, gene transcription and microtubule cytoskeleton organization involved in mitosis (Figure 4A). The KEGG pathway analysis showed circHIPK3 was related to regulating Thyroid hormone signaling, Ras pathway, and ErbB signaling (Figure 4B).

### Silencing of CircHIPK3 Suppressed Glioma Cells Proliferation, Invasion and Migration

**FIGURE 5:**
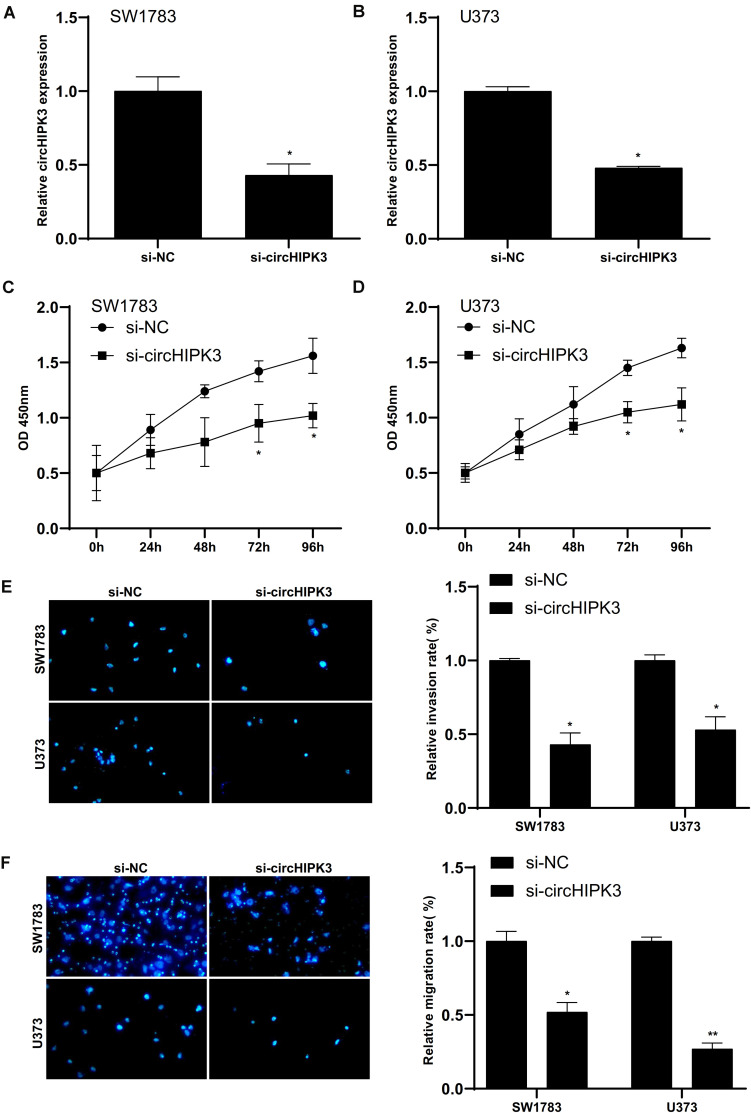
Silencing of circHIPK3 suppressed glioma cells proliferation, invasion and migration. **(A,B)** Silencing efficiency of small interfering RNA against circHIPK3. **(C–F)** CircHIPK3 knockdown markedly suppressed the cell proliferation **(C,D)**, invasion **(E)** and migration **(F)** of glioma cells. ^∗^*p* < 0.05, ^∗∗^*p* < 0.01.

Then, we aimed to explore the roles of circHIPK3 by knocking down of circHIPK3 via transfecting si-circHIPK3. The silence efficiency was showed in Figures 5A,B). The impacts of circHIPK3 silencing on cell proliferation, invasion, and migration on SW1783 and U373 cells was further validated. The results indicated circHIPK3 knockdown markedly suppressed the cell proliferation (Figures 5C,D), invasion (Figure 5E) and migration (Figure 5F) of glioma cells.

### CircHIPK3 Affected CCND2 Expression by Directly Targeting miR-124

**FIGURE 6:**
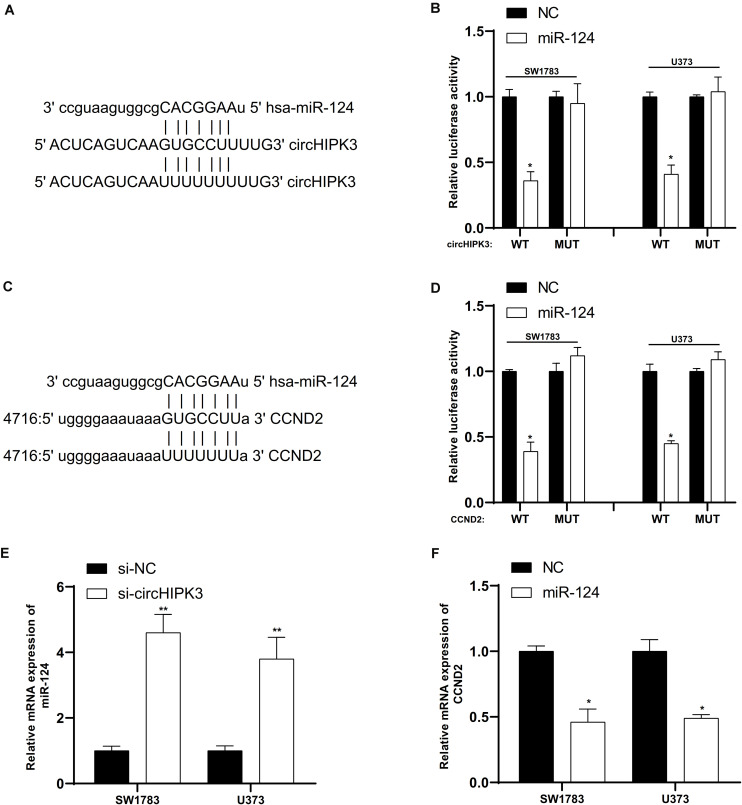
CircHIPK3 affected CCND2 expression by directly targeting miR-124. **(A)** CircHIPK3 had a potential role to sponge miR-124. **(B)** MiR-124 up-regulation markedly suppressed the luciferase activity when co-transfected miR-124 with circHIPK3 wild-type vectors.**(C)** CCND2 was a potential target of miR-124. **(D)** MiR-124 overexpression markedly suppressed the luciferase activity when co-transfected miR-124 with CCND2 wild-type vectors. **(E)** MiR-124 RNA levels were induced after knockdown of circHIPK3. **(F)** MiR-124 overexpression repressed the expression of CCND2. ^∗^*p* < 0.05, ^∗∗^*p* < 0.01.

Based on bioinformatics prediction using online database, circHIPK3 had a potential role to sponge miR-124 (Figure 6A) and CCND2 was a potential target of miR-124 (Figure 6C). To confirm the online predictions, we performed luciferase reporter assays in glioma cells. As illustrated in Figure 6, we observed that miR-124 up-regulation markedly suppressed the luciferase activity when co-transfected miR-124 with circHIPK3 or CCND2 wild-type vectors (Figures 6B,D). Moreover, miR-124 RNA levels were induced after knockdown of circHIPK3 (Figure 6E). However, miR-124 overexpression repressed the expression of CCND2 (Figure 6F).

### The Roles of CircHIPK3 Were Mediated by miR-124 in Glioma Cells

**FIGURE 7:**
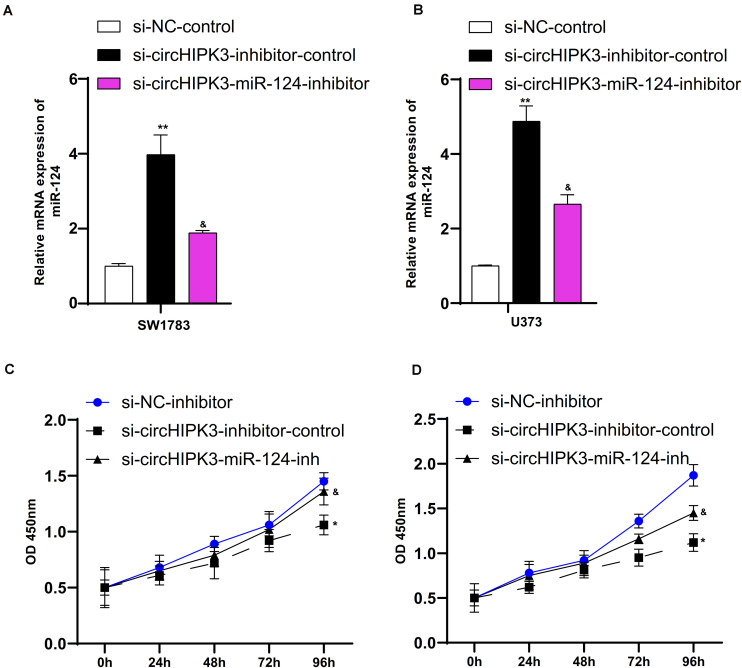
The roles of circHIPK3 were mediated by miR-124 in glioma cells. **(A,B)** MiR-124 expression suppressed in SW1783 and U373 cells using miR-124 inhibitor. **(C,D)** Silencing miR-124 could reverse the degree of optical density (OD) reduction in cells induced by si-circHIPK3 at 450 nm. ^∗^*p* < 0.05, ^∗∗^*p* < 0.01. The sign “&” indicates significant difference between non-control groups, *p* < 0.05.

In order to elucidate the effects of miR-124 on the proliferation of glioma cells, we suppressed miR-124 expression in SW1783 and U373 cells using miR-124 inhibitor (Figures 7A,B). We found the silencing of miR-124 reversed the si-circHIPK3-induced suppressive effects on cell proliferation (Figures 7C,D).

### CCND2 Mediated the Effects of miR-124 on Glioma Cells

**FIGURE 8:**
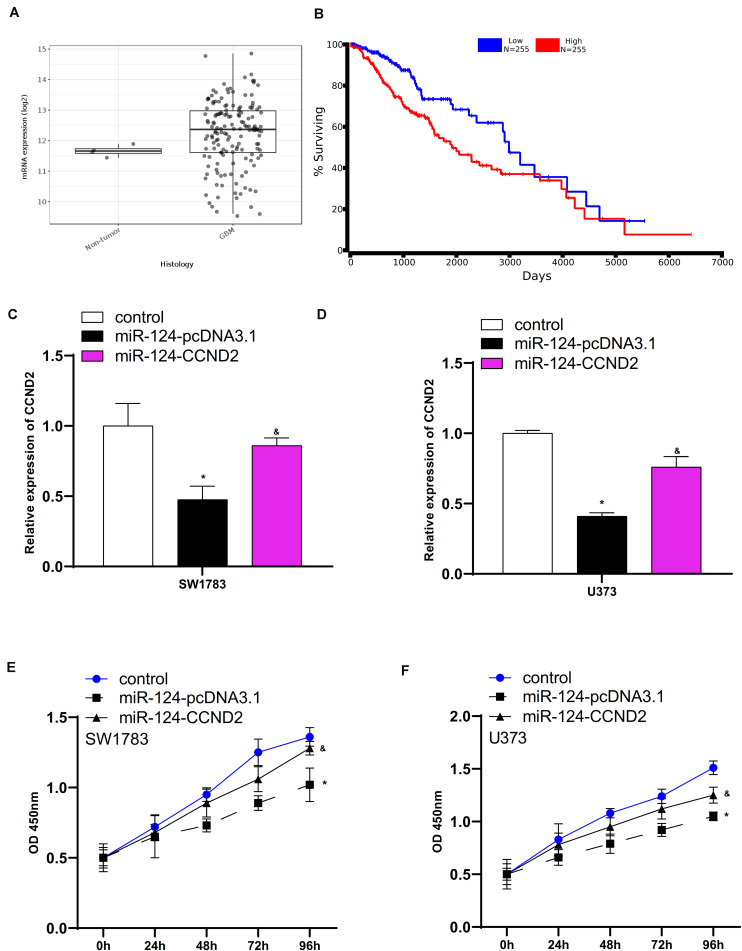
CCND2 mediated the effects of miR-124 on glioma cells. **(A)** CCND2 was up-regulated in glioma samples. **(B)** Correlation between CCND2 expression level and overall survival time of GBM patients. **(C,D)** Overexpression of CCND2 markedly induced CCND2 levels in SW1783 and U373 cells transfected with miR-124. **(E,F)** The reduced cell growth in miR-124 overexpression cells were suppressed by up-regulation of CCND2. ^∗^*p* < 0.05. The sign “&” indicates significant difference between non-control groups, *p* < 0.05.

CCND2, a key cell cycle regulator, has been reported to be an oncogene in human cancers. The TCGA data analysis showed that CCND2 was upregulation in glioma samples compared to normal tissues (Figure 8A). Higher expression levels of CCND2 were correlated to shorter overall survival time in patients with GBM (Figure 8B). To explore whether miR-124 played the tumor suppressive roles in SW1783 and U373 cells by targeting CCND2, we re-expressed CCND2 expression in SW1783 and U373 cells. As presented in Figure 8, overexpression of CCND2 markedly induced CCND2 levels in SW1783 and U373 cells transfected with miR-124 (Figures 8C,D). Further validations showed the reduced cell growth in miR-124 overexpression cells was suppressed by up-regulation of CCND2 (Figures 8E,F).

## Discussion

CircRNAs are a class of stable, abundant RNAs found in the human transcriptome. And unlike ordinary linear RNAs, circRNAs are more conserved and stable because they form a closed loop structure by connecting the 3′ and 5′ ends (Chen and Yang, 2015). CircRNA can act as a miRNA sponge and bind to RNA-binding proteins, thereby targeting miRNAs to perform their functions (Rong et al., 2017). More and more recent evidence indicates that circRNA is involved in a series of pathways and human diseases, including cancer, cardiovascular disease and neurological diseases (Chen et al., 2017). Of course, there are some findings suggesting that the abnormal expression of circRNA is related to the progression of glioma (Hao et al., 2019). Compared to control cells and tissues, the expression of circ-0014359 was increased in glioma cell lines and tissues. The silence of circ-0014359 effectively suppressed the viability, apoptosis and invasion of glioma cells. These indicated that upregulation of circ-0014359 in gliomas was associated with cancer progression.

Bioinformatics analysis showed abundant circHIPK3 modulated cell proliferation via sponging various miRNAs in tumors (Zheng et al., 2016). CircHIPK3 is an oncogene that is up-regulated in CRC, suggesting a poor prognosis (Xie et al., 2020). Moreover, circHIPK3 could sponge and suppress the activity of miR-7, leading to increased expression of FAK (Zeng et al., 2018). CircHIPK3 expression is higher in epithelial ovarian cancer samples than that in adjacent normal tissues (Shabaninejad et al., 2019). High expression of circHIPK3 is correlated to lymph node infiltration and poor prognosis. Therefore, circHIPK3 may be a novel biomarker for predicting EOC prognosis (Liu et al., 2018). CircHIPK3 silencing inhibited the growth of nasopharyngeal carcinoma (NPC) cells *in vitro*. Besides, the absence of circHIPK3 significantly inhibited tumor proliferation. Mechanisms analysis showed circHIPK3 was a miRNA sponge of miR-4288, targeting the ELF3 in NPC cells. Moreover, rescue assays showed circHIPK3 enhanced the malignant behavior of NPC cells by inhibiting miR-4288 and increasing the expression of ELF3, suggesting that circHIPK3 might be a potential target for NPC (Ke et al., 2019). In this study, we found a significant up-regulation in circHIPK3 in glioma tissues and cell lines compared to normal. The silencing of circHIPK3 suppressed the glioma cell proliferation and metastasis.

According to existing research, circular RNA can be interacted with miRNAs and act as a miRNA sponge, thereby regulating downstream genes, thereby promoting the development of many cancers (He et al., 2017). As previously discussed, circHIPK3 can also exert its carcinogenic and tumor-suppressive functions through sponging miRNAs. Analysis showed that miR-124 might be the sponge of circHIPK3. MiR-124 is a miRNA that is specifically expressed in the adult brain and has special potential to determine neural fate (Åkerblom et al., 2012). MiR-124 is found to be overexpressed in both prenatal and postnatal neuronal differentiation. Many scientific research results show that miR-124 can suppress the proliferation of myeloma and adult neural precursors (Maiorano and Mallamaci, 2009). MiR-124 has been shown to be a key progenitor cells differentiation regulator by suppressing PTBP1, which is a brain-specific inhibitor of pre-mRNA splicing (Makeyev et al., 2007). A recent study emphasized that miR-124 induced neurite elongation by inhibiting HDAC5 (Gu et al., 2018). However, the expression and molecular roles of miR-124 has not been explored in glioma. Nor does its underlying mechanism reveal. The present study detected the expression level and molecular function of miR-124 in glioma. We also investigated the potential mechanisms and found that miR-124 regulated glioma through the regulation of CCND2.

CCND2 is a cyclin that is expressed in many tumors and is a common cancer promoter in humans (Büschges et al., 1999). The expression of CCND2 RNA and protein was increased in colorectal cancer (CRC). Moreover, CCND2 expression inhibited by RNA interference suppressed CRC proliferation and migration (Park et al., 2019). In ovarian cancer cells, CCND2 has been proven to be a target of miR-145, and the recovery of this gene partially reverses the effect of miR-145 (Hua et al., 2019). In this study, we found that CCND2 expression was increased in gliomas and was a direct target of miR-124. Moreover, CCND2 overexpression could significantly prevent the reduction of cell proliferation, invasion, and migration caused by miR-124 overexpression. Reversely, miR-124 overexpression repressed the expression of CCND2. What’s more, miR-124 RNA levels were induced after knockdown of circHIPK3. The roles of circHIPK3 were mediated by miR-124 in glioma cells. All in all, circHIPK3 affected CCND2 expression by targeting miR-124, which revealed the correlation among circHIPK3, miR-124 and CCND2.

However, our study still has many limitations. In the present research results, there are many worthy of further study, however, due to the limited energy we have not done enough research. It is also a pity that the conclusion of this paper has not been verified more accurately by animal model experiments. First, the expression level of circHIPK3, miR-124 and CCND2 were not validated using clinical samples. Thus, we should collect more clinical glioma samples and validate the correlation among them in the near future. Second, the *in vivo* assay should be further conducted to demonstrate functional importance of these genes in glioma.

## Conclusion

In summary, this study demonstrates that circHIPK3 is overexpressed in gliomas, and the knockdown of circHIPK3 can reduce the growth capacity of glioma cells, which indicates that its overexpression level promotes gliomas transfer of diffusion. Mechanistically, the interrelationships and interactions between circHIPK3, miR-124, and CCND2 are revealed. In conclusion, this discovery provides a new treatment strategy for gliomas.

## Data Availability Statement

The datasets used and/or analyzed during the current study are available from the corresponding author on reasonable request.

## Ethics Statement

This study was approved by the Medical Ethical Committee of the First Affiliated Hospital of Zhengzhou University. The patients/participants provided their written informed consent to participate in this study. Written informed consent was obtained from the individual(s) for the publication of any potentially identifiable images or data included in this article.

## Author Contributions

ZL: conception and design of the research. SG: acquisition of data. HS: analysis and interpretation of data. YB: statistical analysis. ZS: drafting the manuscript. XL: revision of manuscript for important intellectual content. All authors contributed to the article and approved the submitted version.

## Conflict of Interest

The authors declare that the research was conducted in the absence of any commercial or financial relationships that could be construed as a potential conflict of interest.

## Footnotes

1 https://david.ncifcrf.gov/summary.jsp

2 https://circinteractome.nia.nih.gov/
